# Health-Care Waste Treatment Technology Selection Using the Interval 2-Tuple Induced TOPSIS Method

**DOI:** 10.3390/ijerph13060562

**Published:** 2016-06-04

**Authors:** Chao Lu, Jian-Xin You, Hu-Chen Liu, Ping Li

**Affiliations:** 1 School of Management, Shanghai University, Shanghai 200444, China; chaolu@shu.edu.cn; 2 School of Economics and Management, Tongji University, Shanghai 200092, China; yjx2256@vip.sina.com; 3 Zhoupu Hospital Affiliated to Shanghai University of Medicine & Health Sciences, Shanghai 201318, China; yiwuchulp@126.com

**Keywords:** health-care waste management, interval 2-tuple, TOPSIS, distance measures, HCW treatment technology

## Abstract

Health-care waste (HCW) management is a major challenge for municipalities, particularly in the cities of developing nations. Selecting the best treatment technology for HCW can be regarded as a complex multi-criteria decision making (MCDM) issue involving a number of alternatives and multiple evaluation criteria. In addition, decision makers tend to express their personal assessments via multi-granularity linguistic term sets because of different backgrounds and knowledge, some of which may be imprecise, uncertain and incomplete. Therefore, the main objective of this study is to propose a new hybrid decision making approach combining interval 2-tuple induced distance operators with the technique for order preference by similarity to an ideal solution (TOPSIS) for tackling HCW treatment technology selection problems with linguistic information. The proposed interval 2-tuple induced TOPSIS (ITI-TOPSIS) can not only model the uncertainty and diversity of the assessment information given by decision makers, but also reflect the complex attitudinal characters of decision makers and provide much more complete information for the selection of the optimum disposal alternative. Finally, an empirical example in Shanghai, China is provided to illustrate the proposed decision making method, and results show that the ITI-TOPSIS proposed in this paper can solve the problem of HCW treatment technology selection effectively.

## 1. Introduction

With the rising awareness of the environmental implications of waste disposal, the management and treatment of health-care wastes (HCWs) are gaining more attention all around the world [1,2,3,4]. HCW that is improper handled and disposed of may cause environmental pollution and health problems in terms of proliferation of diseases caused by viruses and micro-organisms, as well as contamination of ground water by untreated medical waste in landfills [5,6]. The HCW management is especially important in developing nations due to conspicuously inappropriate disposal methods, and insufficient financing and infrastructural challenges [7,8,9]. The HCW is defined as all waste materials generated by health care facilities, such as hospitals, clinics, private surgeries, diagnostic centers, dental practices, blood banks, as well as research facilities and laboratories [5,10]. It includes sharps, human tissue, body parts, diagnostic samples, blood, chemicals, pharmaceuticals, medical devices and radioactive materials [10,11]. To avoid human health and environmental issues accompanying poor management of the HCW, both governments and scholars search for effective waste treatment strategies and solutions. Further on, the problems associated with treatment of HCWs should be solved in a manner that minimizes the risks to the public health and human well-being, and the damage to the environment. Therefore, the development of logical and systematic scientific methods is essential to assist urban decision makers in prioritizing and selecting an optimized HCW treatment technology. 

The selection of HCW treatment alternatives is a major complex problem which could be dealt through a multi-criteria analysis. To date, much progress has been made in research relating to the HCW disposal technology selection and a variety of decision support methods have been developed in the literature. For example, Dursun *et al.* [12] proposed a fuzzy multi-criteria decision making (MCDM) method based on the principles of fuzzy measure and fuzzy integral to identify the most suitable HCW treatment alternative for Istanbul. Karagiannidis *et al.* [13] applied analytic hierarchy process (AHP) technique for the effective planning and integrated assessment of scenarios on thermal processing of infectious hospital wastes, and Brent *et al.* [14] adopted the AHP method to establish HCW management systems that minimize infection risks in developing nations. Dursun *et al.* [15] developed two MCDM frameworks using multi-level hierarchical structure and fuzzy set theory for the multi-attribute assessment of HCW disposal alternatives. Liu *et al.* [16] presented a VIKOR-based fuzzy MCDM method for ranking HCW treatment technologies, in which linguistic terms are employed to assess the feasible disposal options and the ordered weighted averaging (OWA) operator is used to aggregate the individual assessments of decision makers. In addition, Liu *et al.* [17] evaluated HCW disposal strategies by applying a modified MULTIMOORA method called interval 2-tuple linguistic MULTIMOORA, and Liu *et al.* [18] selected the appropriate HCW treatment alternative based on a hybrid MCDM model integrating 2-tuple DEMATEL technique and fuzzy MULTIMOORA method. Ciplak [19] identified the best available HCW management option in the Turkish West Black Sea Region with the assistance of a multi-criteria decision analysis framework.

The technique for order preference by similarity to an ideal solution (TOPSIS) method [20] is one of the most classical MCDM methods used for dealing with the selection of the optimal one from a set of available alternatives. It provides an effective framework to investigate complex decision problems based on the evaluation of multiple conflict criteria. The basic concept of the TOPSIS is that the chosen alternative should have the shortest distance from the positive-ideal solution and the longest distance from the negative-ideal solution. Due to its simplicity and ability to yield an indisputable ranking order, the TOPSIS method has been studied and applied in solving a variety of MCDM problems including robot selection [21,22], green supply chain management [23], material selection [24], health-care system evaluation [25] and many other areas of management decision problems [26,27,28]. 

On the other hand, it is usually assumed that the available information is clearly known and can be assessed with exact numbers in the existing HCW treatment technology selection methods. In many situations, however, the input arguments may take the form of linguistic terms because of time pressure, lack of knowledge, and decision makers’ limited attention and information processing capabilities [15,17,18]. Furthermore, decision makers tend to use different linguistic term sets for expressing their evaluations on the established selection criteria considering their personal backgrounds, preferences and different understanding levels to the HCW disposal alternatives. The interval 2-tuple linguistic representation model [29] is highly useful in depicting uncertainty and vagueness of an object, and can be used as a powerful tool to express decision information under various uncertain environments [30,31,32,33]. 

Based upon the above analyses, it can be seen that it is promising to extend the TOPSIS method to accommodate the interval 2-tuple linguistic environment in order to solve the problem of HCW treatment technology selection more efficiently. Therefore, the aim of this paper is to develop a new hybrid decision making approach based on interval 2-tuple linguistic variables and a modified TOPSIS method to select the best disposal technology for HCW management. In particular, we will introduce a distance aggregation operator called the interval 2-tuple induced ordered weighted distance (ITIOWD) operator to improve the TOPSIS method, which parameterizes a wide range of interval 2-tuple distance operators based on a complex reordering process. The proposed interval 2-tuple induced TOPSIS (ITI-TOPSIS) approach can not only model the uncertainty and diversity of assessment information provided by decision makers, but also include the complex attitudinal character of the decision maker in the aggregation process and provide a more complete picture of the HCW treatment technology selection problem. Finally, for the purpose of proving the validity of the proposed method, a case study of selecting the optimum solution for HCW management in Shanghai, China is presented.

The rest of this paper is structured as follows: in Section 2, we introduce some basic concepts regarding interval 2-tupes and present the ITIOWD operator as well as its extensions. In Section 3, we propose the ITI-TOPSIS method for HCW treatment technology selection based on the developed interval 2-tuple induced distance operators. A numerical example is given in Section 4 to demonstrate the proposed approach and to show its feasibility and practicality. Finally, some conclusions and future directions are provided in Section 5.

## 2. Interval 2-Tuples and Induced Distance Operators

### 2.1. 2-Tuple Linguistic Variables

The 2-tuple linguistic representation model was first presented by Herrera and Martínez [34] based on the concept of symbolic translation. To deal with 2-tuple linguistic information under multi-granular linguistic context, Tai and Chen [35] proposed a generalized 2-tuple linguistic model, which can be defined as follows:

**Definition 1.**  Let $S=\left\{s_{0},s_{1},...,s_{g}\right\}$ be a linguistic term set and β∈[0,1] a value representing the result of a symbolic aggregation operation. Then the generalized translation function _∆_ used to obtain the 2-tuple linguistic variable equivalent to β can be defined as follows [35]:

(1) $$\Delta :\left[0,1\right]\to S\times \left[-\frac{1}{2g},\frac{1}{2g}\right)$$

(2) $$\Delta \left(\beta \right)=\left(s_{i},\alpha \right),\;\text{with}\;\{\begin{array}{l} s_{i},\;i=\text{round}(\beta \cdot g)\\ \alpha =\beta -\frac{i}{g},\;\alpha \in \left[-\frac{1}{2g},\frac{1}{2g}\right) \end{array}$$

where round(⋅) is the usual rounding operation, *s_i_* has the closest index label to β and α is the value of the symbolic translation.

**Definition 2.**  Let $S=\left\{s_{0},s_{1},...,s_{g}\right\}$ be a linguistic term set and $\left(s_{i},\alpha \right)$ be a 2-tuple. There exists a function $\Delta^{-1}$, which is able to convert a 2-tuple linguistic variable into its equivalent numerical value β∈[0,1]. The reverse function $\Delta^{-1}$ is defined as follows [35]:

(3) $$\Delta^{-1}:S\times \left[-\frac{1}{2g},\frac{1}{2g}\right)\to \left[0,1\right]$$

(4) $$\Delta^{-1}\left(s_{i},\alpha \right)=\frac{i}{g}+\alpha =\beta$$

The conversion of a linguistic term into a linguistic 2-tuple consists of adding a value 0 as symbolic translation [34]:

(5) $$s_{i}\in S\Rightarrow \left(s_{i},0\right)$$

**Definition 3.**  Let $\left(s_{k},\alpha_{1}\right)$ and $\left(s_{l},\alpha_{2}\right)$ be two 2-tuples, then the comparison of linguistic information represented by 2-tuples is carried out according to the following rules [34]:

(1) If *k* < *l* then $\left(s_{k},\alpha_{1}\right)$ is smaller than $\left(s_{l},\alpha_{2}\right)$;
(2) If *k* = *l* then
  (a) If *α*_1_ = *α*_2_, then $\left(s_{k},\alpha_{1}\right)$ is equal to $\left(s_{l},\alpha_{2}\right)$;
  (b) If *α*_1_ < *α*_2_, then $\left(s_{k},\alpha_{1}\right)$ is smaller than $\left(s_{l},\alpha_{2}\right)$;
  (c) If *α*_1_> *α*_2_, then $\left(s_{k},\alpha_{1}\right)$ is bigger than $\left(s_{l},\alpha_{2}\right)$.

### 2.2. Interval 2-Tuple Linguistic Variables

Based on the 2-tuple linguistic variables, Zhang [29] further introduced the interval 2-tuple linguistic representation model to better express decision information.

**Definition 4.**  Let $S=\left\{s_{0},s_{1},...,s_{g}\right\}$ be a linguistic term set. An interval 2-tuple linguistic variable is composed of two 2-tuples, denoted by $\left[\left(s_{k},\alpha_{1}\right),\left(s_{l},\alpha_{2}\right)\right]$, where $\left(s_{k},\alpha_{1}\right)\leq \left(s_{l},\alpha_{2}\right)$. Then the interval 2-tuple that expresses equivalent information to an interval value $\left[\beta_{1},\beta_{2}\right]\left(\beta_{1},\beta_{2}\in \left[0,\;1\right],\beta_{1}\leq \beta_{2}\right)$ is derived by the following function [29]:

(6) $$\Delta \left[\beta_{1},\beta_{2}\right]=\left[\left(s_{k},\alpha_{1}\right),\left(s_{l},\alpha_{2}\right)\right]\;\text{with}\;\{\begin{array}{l} s_{k},\;k=\text{round}\left(\beta_{1}\cdot g\right)\\ s_{l},\;l=\text{round}\left(\beta_{2}\cdot g\right)\\ \alpha_{1}=\beta_{1}-\frac{k}{g},\;\alpha_{1}\in \left[-\frac{1}{2g},\frac{1}{2g}\right)\\ \alpha_{2}=\beta_{2}-\frac{l}{g},\;\alpha_{2}\in \left[-\frac{1}{2g},\frac{1}{2g}\right) \end{array}$$

Specially, if $\left(s_{k},\alpha_{1}\right)=\left(s_{l},\alpha_{2}\right)$, then the interval 2-tuple linguistic variable reduces to a 2-tuple linguistic variable.

On the contrary, there is always a function $\Delta^{-1}$ such that an interval 2-tuple can be converted into an interval value $\left[\beta_{1},\beta_{2}\right]\left(\beta_{1},\beta_{2}\in \left[0,\;1\right],\beta_{1}\leq \beta_{2}\right)$ as follows:

(7) $$\Delta^{-1}\left[\left(s_{k},\alpha_{1}\right),\left(s_{l},\alpha_{2}\right)\right]=\left[\frac{k}{g}+\alpha_{1},\frac{l}{g}+\alpha_{2}\right]=\left[\beta_{1},\beta_{2}\right]$$

Consider any three interval 2-tuples $\tilde{a}=\left[\left(r,\alpha \right),\left(t,\varepsilon \right)\right],$ ${\tilde{a}}_{1}=\left[\left(r_{1},\alpha_{1}\right),\left(t_{1},\varepsilon_{1}\right)\right]$ and ${\tilde{a}}_{2}=\left[\left(r_{2},\alpha_{2}\right),\left(t_{2},\varepsilon_{2}\right)\right]$, and let λ∈[0, 1], then their operations are defined as follows [36]:

(1) ${\tilde{a}}_{1}⊕{\tilde{a}}_{2}=\left[\left(r_{1},\alpha_{1}\right),\left(t_{1},\varepsilon_{1}\right)\right]⊕\left[\left(r_{2},\alpha_{2}\right),\left(t_{2},\varepsilon_{2}\right)\right]$ $=\Delta \left[\Delta^{-1}\left(r_{1},\alpha_{1}\right)+\Delta^{-1}\left(r_{2},\alpha_{2}\right),\Delta^{-1}\left(t_{1},\varepsilon_{1}\right)+\Delta^{-1}\left(t_{2},\varepsilon_{2}\right)\right];$
(2) $\lambda \tilde{a}=\lambda \left[\left(r,\alpha \right),\left(t,\varepsilon \right)\right]=\Delta \left[\lambda \Delta^{-1}\left(r,\alpha \right),\lambda \Delta^{-1}\left(t,\varepsilon \right)\right].$

**Definition 5.**  Let ${\tilde{a}}_{i}=\left[\left(r_{i},\alpha_{i}\right),\left(t_{i},\varepsilon_{i}\right)\right]\left(i=1,2,...,n\right)$ be a set of interval 2-tuples and $w={\left(w_{1},w_{2},...,w_{n}\right)}^{T}$ be their associated weights, with $w_{i}\in \left[0,\;1\right],\sum_{i=1}^{n}w_{i}=1$. The interval 2-tuple weighted average (ITWA) operator is defined as [29]:

(8) $$\begin{array}{cc} {\text{ITWA}}_{w} & \left({\tilde{a}}_{1},{\tilde{a}}_{2},...,{\tilde{a}}_{n}\right)=\overset{n}{\underset{i=1}{⊕}}\left(w_{i}{\tilde{a}}_{i}\right)\\ & =\Delta \left[\sum_{i=1}^{n}w_{i}\Delta^{-1}\left(s_{i},\alpha_{i}\right),\sum_{i=1}^{n}w_{i}\Delta^{-1}\left(t_{i},\varepsilon_{i}\right)\right] \end{array}$$

**Definition 6.**  Let ${\tilde{a}}_{1}=\left[\left(r_{1},\alpha_{1}\right),\left(t_{1},\varepsilon_{1}\right)\right]$ and ${\tilde{a}}_{2}=\left[\left(r_{2},\alpha_{2}\right),\left(t_{2},\varepsilon_{2}\right)\right]$ be two interval 2-tuples, then:

(9) $$d_{ITD}\left({\tilde{a}}_{1},{\tilde{a}}_{2}\right)=\Delta \left[\frac{1}{2}\left(\left|\Delta^{-1}\left(r_{1},\alpha_{1}\right)-\Delta^{-1}\left(r_{2},\alpha_{2}\right)\right|+\left|\Delta^{-1}\left(t_{1},\varepsilon_{1}\right)-\Delta^{-1}\left(t_{2},\varepsilon_{2}\right)\right|\right)\right]$$

is called the interval 2-tuple distance between $\tilde{a}$ and $\tilde{b}$.

### 2.3. Interval 2-Tuple Induced Distance Operators

Inspired by the induced ordered weighted averaging distance (IOWAD) operator [37], in what follows, we define an interval 2-tuple induced ordered weighted distance (ITIOWD) operator. Let $\tilde{S}$ be the set of all interval 2-tuples, $\hat{S}$ be the set of all 2-tuples, $\tilde{A}=\left\{{\tilde{a}}_{1},{\tilde{a}}_{2},...,{\tilde{a}}_{n}\right\}$ and $\tilde{B}=\left\{{\tilde{b}}_{1},{\tilde{b}}_{2},...,{\tilde{b}}_{n}\right\}$ be two sets of interval 2-tuples, it can be defined in the following way:

**Definition 7.**  An ITIOWD operator of dimension *n* is a mapping ITIOWD: $R^{n}\times {\tilde{S}}^{n}\times {\tilde{S}}^{n}\to \hat{S}$ that has an associated weighting vector $\omega ={\left(\omega_{1},\omega_{2},...,\omega_{n}\right)}^{T}$, with $\omega_{j}\in \left[0,\;1\right]$ and $\Sigma_{j=1}^{n}\omega_{j}=1$, such that:

(10) $$\text{ITIOWD}\left(\langle u_{1},{\tilde{a}}_{1},{\tilde{b}}_{1}\rangle ,\langle u_{2},{\tilde{a}}_{2},{\tilde{b}}_{2}\rangle ,...,\langle u_{n},{\tilde{a}}_{n},{\tilde{b}}_{n}\rangle \right)=\sum_{j=1}^{n}\omega_{j}{\hat{d}}_{j}$$

where ${\hat{d}}_{j}$ represents the $d_{ITD}\left({\tilde{a}}_{i},{\tilde{b}}_{i}\right)$ value of the ITIOWD triplet $\langle u_{i},{\tilde{a}}_{i},{\tilde{b}}_{i}\rangle$ having the *j*th largest $u_{i}$, $u_{i}$ is the order inducing variable and $d_{ITD}\left({\tilde{a}}_{i},{\tilde{b}}_{i}\right)$ is the argument variable represented in the form of individual interval 2-tuple distance.

Especially, if there is a tie between $\langle u_{i},{\tilde{a}}_{i},{\tilde{b}}_{i}\rangle$ and $\langle u_{j},{\tilde{a}}_{j},{\tilde{b}}_{j}\rangle$ with respect to the order inducing variables such that *u_i_* = *u_j_*, we replace the argument component of each of $\langle u_{i},{\tilde{a}}_{i},{\tilde{b}}_{i}\rangle$ and $\langle u_{j},{\tilde{a}}_{j},{\tilde{b}}_{j}\rangle$ by their interval 2-tuple normalized distance $\left(d_{ITD}\left({\tilde{a}}_{i},{\tilde{b}}_{i}\right)+d_{ITD}\left({\tilde{a}}_{j},{\tilde{b}}_{j}\right)\right)/2$ in the process of aggregation. If the sets of interval 2-tuples $\tilde{A}=\left\{{\tilde{a}}_{1},{\tilde{a}}_{2},...,{\tilde{a}}_{n}\right\}$ and $\tilde{B}=\left\{{\tilde{b}}_{1},{\tilde{b}}_{2},...,{\tilde{b}}_{n}\right\}$ are degenerated to the sets of 2-tuples $\hat{A}=\left\{{\hat{a}}_{1},{\hat{a}}_{2},...,{\hat{a}}_{n}\right\}$ and $\hat{B}=\left\{{\hat{b}}_{1},{\hat{b}}_{2},...,{\hat{b}}_{n}\right\}$, then the ITIOWD is reduced to the 2-tuple induced ordered weighted distance (TIOWD) operator:

(11) $$\text{TIOWD}\left(\langle u_{1},{\hat{a}}_{1},{\hat{b}}_{1}\rangle ,...,\langle u_{n},{\hat{a}}_{n},{\hat{b}}_{n}\rangle \right)=\sum_{j=1}^{n}\omega_{j}{\hat{d}}_{j}$$

where ${\hat{d}}_{j}$ represents the 2-tuple distance $d_{TD}\left({\hat{a}}_{i},{\hat{b}}_{i}\right)$ of the TIOWD triplet $\langle u_{i},{\hat{a}}_{i},{\hat{b}}_{i}\rangle$ having the *j*th largest $u_{i}$, $u_{i}$ is the order inducing variable and $d_{TD}\left({\hat{a}}_{i},{\hat{b}}_{i}\right)$ is the argument variable represented in the form of individual 2-tuple distance.

Similar to the IOWAD operator [37], the ITIOWD operator is commutative, monotonic, bounded, idempotent, and non-negative. In the literature, a lot of methods have been suggested for determining the OWA weights, which can also be implemented for the ITIOWD operator. In this study, to relieve the influence of unfair arguments on the decision results, the normal distribution-based method suggested by Xu [38] is used to generate the weights of the ITIOWD operator.

The ITIOWD operator provides a parameterized family of interval 2-tuple distance operators by a different manifestation of the weighting vector *ω*. For example, with the ITIOWD operator, the maximum interval 2-tuple distance is found if $\omega_{k}=1$ and $\omega_{j}=0$, for all j≠k, and $u_{k}=\max\left\{d_{ITD}\left({\tilde{a}}_{i},{\tilde{b}}_{i}\right)\right\}$. The minimum interval 2-tuple distance is found when $\omega_{k}=1$ and $\omega_{j}=0$, for all j≠k, and $u_{k}=\min\left\{d_{ITD}\left({\tilde{a}}_{i},{\tilde{b}}_{i}\right)\right\}$. The interval 2-tuple normalized Hamming distance (ITNHD) operator is obtained if $\omega_{j}=1/n$ for all *j*. The interval 2-tuple weighted Hamming distance (ITWHD) operator is found if the ordered position of *μ_i_* is the same as the position of $d_{ITD}\left({\tilde{a}}_{i},{\tilde{b}}_{i}\right)$. The interval 2-tuple ordered weighted distance (ITOWD) is formed if the ordered position of *μ_i_* is the same than the ordered position of $d_{ITD}\left({\tilde{a}}_{i},{\tilde{b}}_{i}\right)$.

In what follows, generalizations of the ITIOWD operator are presented by using the generalized and the quasi-arithmetic means. We define the generalized ITIOWD (GITIOWD) operator and the quasi-arithmetic ITIOWD (Quasi-ITIOWD) operator.

**Definition 8.**  A GITIOWD operator of dimension *n* is a mapping $\mathrm{GITIOWD}:\;R^{n}\times {\tilde{S}}^{n}\times {\tilde{S}}^{n}\to \hat{S}$ that has an associated weighting vector $\omega ={\left(\omega_{1},\omega_{2},...,\omega_{n}\right)}^{T}$, with $\omega_{j}\in \left[0,\;1\right]$ and $\Sigma_{j=1}^{n}\omega_{j}=1$, such that:

(12) $$\text{GITIOWD}\left(\langle u_{1},{\tilde{a}}_{1},{\tilde{b}}_{1}\rangle ,...,\langle u_{n},{\tilde{a}}_{n},{\tilde{b}}_{n}\rangle \right)={\left(\sum_{j=1}^{n}\omega_{j}{\hat{d}}_{j}^{\lambda }\right)}^{1/\lambda }$$

where ${\hat{d}}_{j}$ represents the $d_{ITD}\left({\tilde{a}}_{i},{\tilde{b}}_{i}\right)$ value of the GITIOWD triplet $\langle u_{i},{\tilde{a}}_{i},{\tilde{b}}_{i}\rangle$ having the *j*th largest $u_{i}$, $u_{i}$ is the order inducing variable, $d_{ITD}\left({\tilde{a}}_{i},{\tilde{b}}_{i}\right)$ is the argument variable represented in the form of individual interval 2-tuple distance and λ is a parameter such that λ∈(−∞,+∞)−{0}.

Following [39], we are able to obtain different types of distance operators by analyzing the weighting vector ω and the parameter λ in the GITIOWD operator. For example, we can obtain the following cases:

- If $\omega_{j}=1/n$, for all *j*, we obtain the generalized interval 2-tuple normalized distance (GITND) operator.
- If λ=1, we get the ITIOWD operator.
- If λ=2, we get the interval 2-tuple induced ordered weighted Euclidean distance (ITIOWED) operator.
- If λ→0, we get the interval 2-tuple induced ordered weighted geometric distance (ITIOWGD) operator.
- If λ=−1, we get the interval 2-tuple induced ordered weighted harmonic distance (ITIOWHD) operator.
- If λ=3, we get the interval 2-tuple induced ordered weighted cubic distance (ITIOWCD) operator.

**Definition 9.**  A Quasi-ITIOWD operator of dimension *n* is a mapping Quasi-ITIOWD: $R^{n}\times {\tilde{S}}^{n}\times {\tilde{S}}^{n}\to \hat{S}$, that has an associated weighting vector $\omega ={\left(\omega_{1},\omega_{2},...,\omega_{n}\right)}^{T}$, with $\omega_{j}\in \left[0,\;1\right]$ and $\Sigma_{j=1}^{n}\omega_{j}=1$, such that:

(13) $$\text{Quasi-ITIOWD}\left(\langle u_{1},{\tilde{a}}_{1},{\tilde{b}}_{1}\rangle ,...,\langle u_{n},{\tilde{a}}_{n},{\tilde{b}}_{n}\rangle \right)=g^{-1}\left(\sum_{j=1}^{n}\omega_{j}g\left({\hat{d}}_{j}\right)\right)$$

where ${\hat{d}}_{j}$ represents the $d_{ITD}\left({\tilde{a}}_{i},{\tilde{b}}_{i}\right)$ value of the Quasi-ITIOWD triplet $\langle u_{i},{\tilde{a}}_{i},{\tilde{b}}_{i}\rangle$ having the *j*th largest $u_{i}$, $u_{i}$ is the order inducing variable, $d_{ITD}\left({\tilde{a}}_{i},{\tilde{b}}_{i}\right)$ is the argument variable represented in the form of individual interval 2-tuple distance and *g* is a general continuous strictly monotone function. As we can see, the GITIOWD operator is a particular case of the Quasi-ITIOWD operator when $g\left({\hat{d}}_{j}\right)={\hat{d}}_{j}^{\lambda }$.

## 3. The Proposed ITI-TOPSIS Method for Selecting HCW Technologies

**Figure 1:**
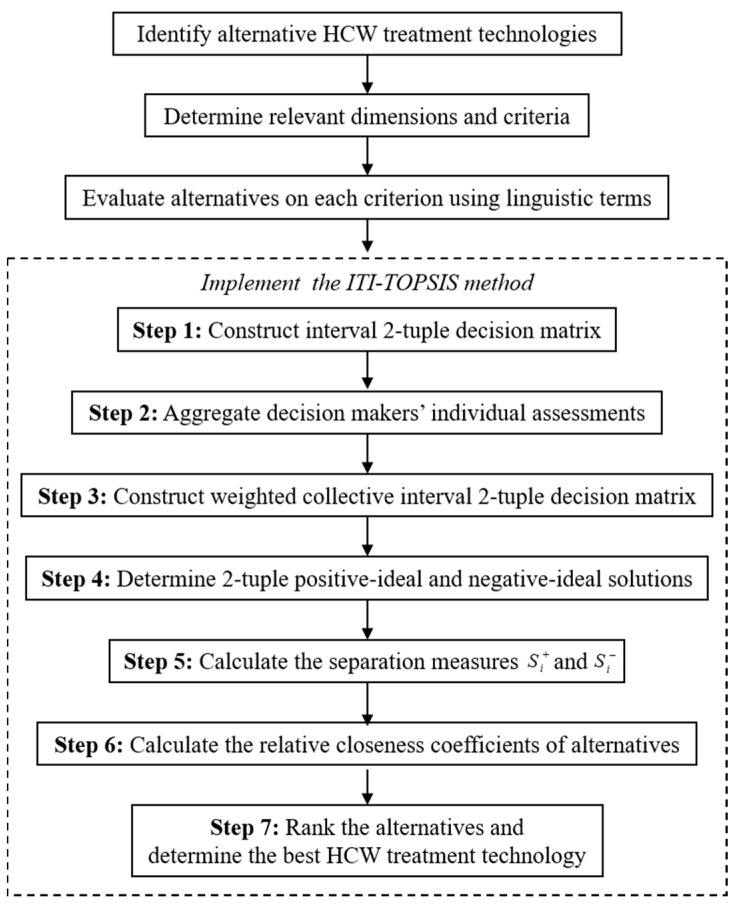
Flowchart of the proposed ITI-TOPSIS method.

HCW management is a high priority environmental concern throughout the world, which can lead to numerous possible health and safety hazards for people and the environment if inadequately treated. In this section, we develop an integrated MCDM framework using interval 2-tuple induced distance operators and an extended TOPSIS method for selecting the best and most effective treatment method for HCW management. The decision process to follow in the HCW treatment technology selection is similar to the classical TOPSIS process developed in [20], with the difference being that the method proposed here will employ the ITIOWD operator to calculate the separate measures of each alternative from the positive-ideal and the negative-ideal solutions. Figure 1 delineates the flowchart of the proposed decision support framework to determine the most appropriate HCW disposal method. 

Suppose that a HCW disposal technology selection problem has *l* decision makers $DM_{k}\left(k=1,\;2,\;...,\;l\right)$, *m* alternatives $A_{i}\left(i=1,\;2,\;...,\;m\right)$, and *n* decision criteria $C_{j}\left(j=1,2,\;...,n\right)$. Each decision maker *DM_k_* is given a weight $v_{k}>0\left(k=1,\;2,\;...,\;l\right)$ satisfying $\sum_{k=1}^{l}v_{k}=1$ to reflect his/her relative importance in the group decision making process. Let $D_{k}={\left(d_{ij}^{k}\right)}_{m\times n}$ be the linguistic decision matrix of the *k*th decision maker, where $d_{ij}^{k}$ is the linguistic information provided by *DM_k_* on the assessment of *A_i_* with respect to C*_j_*. In addition, different linguistic term sets may be used by the decision makers to express their preference values.

Then based on the ITIOWD operator, we propose an interval 2-tuple induced TOPSIS (ITI-TOPSIS) method to resolve HCW treatment technology selection problems with linguistic information. It involves the following steps: 

**Step 1:** Convert the linguistic decision matrix $D_{k}={\left(d_{ij}^{k}\right)}_{m\times n}$ into interval 2-tuple decision matrix ${\tilde{R}}_{k}={\left({\tilde{r}}_{ij}^{k}\right)}_{m\times n}={\left(\left[\left(r_{ij}^{k},0\right),\left(t_{ij}^{k},0\right)\right]\right)}_{m\times n}$, where $r_{ij}^{k},t_{ij}^{k}\in S,S=\left\{s_{0},s_{1},...,s_{g}\right\}$ and $r_{ij}^{k}\leq t_{ij}^{k}$. 

Suppose that *DM_k_* provides his assessments in a set of five linguistic terms and the linguistic term set is expressed as $S=\{s_{0}=Very\;poor,s_{1}=Poor,s_{2}=Medium,s_{3}=Good,s_{4}=Very\;good\}.$ Then the linguistic information provided in the decision matrix *D_k_* can be transformed into its corresponding interval 2-tuple linguistic assessments according to the following ways:

- A certain grade such as *Poor* can be written as $\left[\left(s_{1},0\right),\;\left(s_{1},0\right)\right]$.
- An interval grade such as *Poor-Medium*, which means that the assessment of an alternative with respect to the criterion under consideration is between *Poor* and *Medium*. This can be expressed as $\left[\left(s_{1},0\right),\left(s_{2},0\right)\right]$.

**Step 2:** Utilize the ITWA operator:

(14) $$\begin{array}{cc} {\tilde{r}}_{ij} & =\left[\left(r_{ij},\alpha_{ij}\right),\left(t_{ij},\varepsilon_{ij}\right)\right]\;=\text{ITWA}\left({\tilde{r}}_{ij}^{1},{\tilde{r}}_{ij}^{2},...,{\tilde{r}}_{ij}^{l}\right)\\ & =\Delta \left[\sum_{k=1}^{l}v_{k}\Delta^{-1}\left(r_{ij}^{k},0\right),\sum_{k=1}^{l}v_{k}\Delta^{-1}\left(t_{ij}^{k},0\right)\right],\;\;i=1,\;2,\;...,\;m,j=1,\;2,\;...,\;n \end{array}$$

to aggregate all the interval 2-tuple decision matrices ${\tilde{R}}_{k}\left(k=1,2,...,l\right)$ into a collective interval 2-tuple decision matrix $\tilde{R}={\left({\tilde{r}}_{ij}\right)}_{m\times n}$.

**Step 3:** Construct the weighted collective interval 2-tuple decision matrix ${\tilde{R}}^{\prime }={\left({\tilde{r}}_{ij}^{\prime }\right)}_{m\times n}$ according to the following equation:

(15) $$\begin{array}{cc} {\tilde{r}}_{ij}^{\prime } & =\left[\left(r_{ij}^{\prime },\alpha_{ij}^{\prime }\right),\left(t_{ij}^{\prime },\varepsilon_{ij}^{\prime }\right)\right]\;=w_{j}\left[\left(r_{ij},\alpha_{ij}\right),\left(t_{ij},\varepsilon_{ij}\right)\right]\;\\ & =\Delta \left[w_{j}\Delta^{-1}\left(r_{ij},\alpha_{ij}\right),w_{j}\Delta^{-1}\left(t_{ij},\varepsilon_{ij}\right)\right],\;\;i=1,\;2,\;...,\;m,j=1,\;2,\;...,\;n \end{array}$$

where *w_j_* is the weight of the *j*th criterion, $w_{j}\geq 0,j=1,2,...,n,$ and $\sum_{j=1}^{n}w_{j}=1$.

**Step 4:** Determine the 2-tuple positive ideal solution ${\hat{A}}^{*}=\left({\hat{r}}_{1}^{*},{\hat{r}}_{2}^{*},...,{\hat{r}}_{n}^{*}\right)$ and the 2-tuple negative ideal solution ${\hat{A}}^{-}=\left({\hat{r}}_{1}^{-},{\hat{r}}_{2}^{-},...,{\hat{r}}_{n}^{-}\right)$, where:

(16) $${\hat{r}}_{j}^{*}=\left\{\begin{array}{l} \underset{i}{\max}\left\{\left(t_{ij}^{\prime },\varepsilon_{ij}^{\prime }\right)\right\},\;\text{for}\;\text{benefit criteria}\\ \underset{i}{\min}\left\{\left(r_{ij}^{\prime },\alpha_{ij}^{\prime }\right)\right\},\;\text{for}\;\text{cost criteria} \end{array}\right\},\;\;\;j=1,\;2,\;...,\;n$$

(17) $${\hat{r}}_{j}^{-}=\left\{\begin{array}{l} \underset{i}{\min}\left\{\left(r_{ij}^{\prime },\alpha_{ij}^{\prime }\right)\right\},\;\text{for}\;\text{benefit criteria}\\ \underset{i}{\max}\left\{\left(t_{ij}^{\prime },\varepsilon_{ij}^{\prime }\right)\right\},\;\text{for}\;\text{cost criteria} \end{array}\right\},\;\;\;j=1,\;2,\;...,\;n$$

**Step 5:** Calculate the separation measures, $S_{i}^{+}$ and $S_{i}^{-}$ of each alternative from the 2-tuple positive-ideal and the negative-ideal solutions using the ITIOWD operator:

(18) $$\begin{array}{cc} S_{i}^{*} & =\text{ITIOWD}\left(\langle u_{1},{\tilde{r}}_{i1},{\hat{r}}_{1}^{*}\rangle ,\langle u_{2},{\tilde{r}}_{i2},{\hat{r}}_{2}^{*}\rangle ,...,\langle u_{j},{\tilde{r}}_{ij},{\hat{r}}_{j}^{*}\rangle \right)\\ & =\sum_{j=1}^{n}\omega_{j}{\hat{d}}_{j}^{*},\;\;i=1,\;2,\;...,\;m \end{array}$$

(19) $$\begin{array}{cc} S_{i}^{-} & =\text{ITIOWD}\left(\langle u_{1},{\tilde{r}}_{i1},{\hat{r}}_{1}^{-}\rangle ,\langle u_{2},{\tilde{r}}_{i2},{\hat{r}}_{2}^{-}\rangle ,...,\langle u_{j},{\tilde{r}}_{ij},{\hat{r}}_{j}^{-}\rangle \right)\\ & =\sum_{j=1}^{n}\omega_{j}{\hat{d}}_{j}^{-},\;\;i=1,\;2,\;...,\;m \end{array}$$

where ${\hat{d}}_{j}^{*}$ is the $d_{ITD}\left({\tilde{r}}_{ij},{\hat{r}}_{j}^{*}\right)$ value of the ITIOWD triplet $\langle u_{j},{\tilde{r}}_{ij},{\hat{r}}_{j}^{*}\rangle$ having the *j*th largest $u_{i}$, ${\hat{d}}_{j}^{-}$ is the $d_{ITD}\left({\tilde{r}}_{ij},{\hat{r}}_{j}^{-}\right)$ value of the ITIOWD triplet $\langle u_{j},{\tilde{r}}_{ij},{\hat{r}}_{j}^{-}\rangle$ having the *j*th largest $u_{i}$, $u_{i}$ is the order inducing variable, and $\omega ={\left(\omega_{1},\omega_{2},...,\omega_{n}\right)}^{T}$ is the weighting vector of the ITIOWD operator such that $\omega_{j}\in \left[0,\;1\right]$ and $\sum_{j=1}^{n}\omega_{j}=1$. It is important to highlight that different interval 2-tuple induced distance operators can be used in this step, as those described in the previous section.

**Step 6**: Calculate the relative closeness coefficient of each alternative to the 2-tuple ideal solution by:

(20) $$C_{i}^{*}=\frac{S_{i}^{-}}{S_{i}^{*}+S_{i}^{-}}\;\;i=1,\;2,\;...,\;m$$

where $0\leq \Delta^{-1}\left(C_{i}^{*}\right)\leq 1$.

**Step 7:** Rank all the alternatives $A_{i}\left(i=1,\;2,\;...,\;m\right)$ and determine the optimal one(s) according to the descending order of their closeness coefficients. The bigger the value $C_{i}^{*}$, the better the alternative $A_{i}$.

## 4. An Illustrative Example

**Figure 2:**
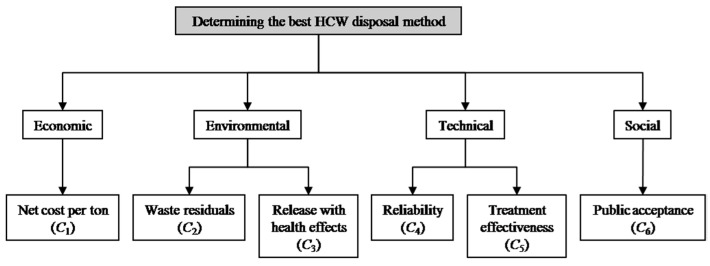
Hierarchical structure of the problem.

In this section, an empirical example conducted in Shanghai, China [16,18] is presented to illustrate the application of the proposed decision support method to the HCW treatment technology selection problem. Shanghai is one of the largest cities in China, with a population of over 24 million people dispersed in 16 different district municipalities. According to the 2015 census results, the population density of the city is 3826 people/km^2^. In view of the training effort and the consequence of the regulation, the amount of HCWs collected and processed at the incineration plants in Shanghai has steadily increased in recent years. The capacities of current incineration plants are not adequate to deal with all the medical wastes generated from healthcare facilities of the city. Therefore, it is now need to determine the best HCW treatment technology for processing the medical wastes with the proposed ITI-TOPSIS procedure. Through interacting and communicating with experts from environmental protection bureau and companies responsible for collecting medical wastes in Shanghai, we reviewed and analyzed the HCW treatment technologies that are currently used in the city, and discussed the problems encountered in HCW management. After preliminary screening, four disposal methods have remained as alternatives for further evaluation, *i.e.*, incineration (*A*_1_), steam sterilization (*A*_2_), microwave (*A*_3_), and landfill (*A*_4_). In order to select the most preferred one, an expert committee of five decision makers, *DM*_1_, *DM*_2_, *DM*_3_, *DM*_4_ and *DM*_5_ has been formed. In addition, based on an extensive literature review regarding the evaluation of HCW disposal alternatives and expert interviews, economic, environmental, technical and social criteria are identified as the selection criteria. Corresponding to these criteria, several relevant sub-criteria are also defined in order to conduct a comprehensive assessment of the HCW treatment technologies. The hierarchical structure of the problem is depicted in Figure 2.

The five decision makers in the expert panel employ different linguistic term sets to assess the suitability of the alternatives with respect to the above evaluation criteria. Specifically, the decision makers *DM*_1_ and *DM*_4_ provide their assessments using the five-label linguistic term set *A*; *DM*_2_ and *DM*_5_ provide their assessments using the seven-point linguistic scale *B*; *DM*_3_ provides his assessments using the nine-label linguistic term set *C*. These linguistic term sets are denoted as follows:

$$\begin{array}{cc} A= & \{a_{0}=Very\;low\left(VL\right),a_{1}=Low\left(L\right),a_{2}=Moderate\left(M\right),\;a_{3}=High\left(H\right),\\ & a_{4}=Very\;high\left(VH\right)\} \end{array}$$

$$\begin{array}{cc} B= & \{b_{0}=Very\;low\left(VL\right),b_{1}=Low\left(L\right),b_{2}=Moderatelow\left(ML\right),b_{3}=Moderate\left(M\right),\\ & b_{4}=Moderate\;high\left(MH\right),b_{5}=High\left(H\right),b_{6}=Very\;high\left(VH\right)\} \end{array}$$

$$\begin{array}{cc} C= & \{c_{0}=Extra\;low\left(EL\right),c_{1}=Very\;low\left(VL\right),c_{2}=Low\left(L\right),c_{3}=Moderate\;low\left(ML\right),\\ & c_{4}=Moderate\left(M\right),c_{5}=Moderate\;high\left(MH\right),c_{6}=High\left(H\right),c_{7}=Very\;high\left(VH\right),\\ & c_{8}=Extra\;high\left(EH\right)\} \end{array}$$

The linguistic assessments of the four alternatives with respect to each criterion given by the five decision makers are presented in Table 1. Due to the complex attitudinal characters of the decision makers, they need to use order inducing variables shown in Table 2 in the reordering process. Since the results given by each alternative are not equal, the decision maker assumes a different attitudinal character for each alternative in this case study. 

With the information obtained, we can utilize the TI-TOPSIS method to derive the most desirable HCW treatment technology. First, we convert the linguistic decision matrix shown in Table 1 into the interval 2-tuple decision matrix ${\tilde{R}}_{k}={\left(\left[\left(r_{ij}^{k},0\right),\left(t_{ij}^{k},0\right)\right]\right)}_{4\times 6}$, which is depicted in Table 3. Then, we aggregate the individual assessments of the five decision makers to obtain a collective interval 2-tuple decision matrix by using the ITWA operator in Equation (14) and to construct a weighted collective interval 2-tuple decision matrix by using Equation (15). The results are shown in Table 4 and Table 5. In this example, the weighting vectors of the five decision makers and the six selection criteria are assumed as $v={\left(0.15,0.20,0.30,0.25,0.10\right)}^{T}$ and $w={\left(0.19,0.16,0.20,0.15,0.18,0.12\right)}^{T}$, respectively. 

Next, net cost per ton (*C_1_*), waste residuals (*C*_2_) and release with health effects (*C*_3_) are cost criteria; reliability (*C*_4_), treatment effectiveness (*C*_5_) and public acceptance (*C*_6_) are benefit criteria. Thus, the 2-tuple positive ideal solution ${\hat{A}}^{*}=\left({\hat{r}}_{1}^{*},{\hat{r}}_{2}^{*},...,{\hat{r}}_{6}^{*}\right)$ and the 2-tuple negative ideal solution ${\hat{A}}^{-}=\left({\hat{r}}_{1}^{-},{\hat{r}}_{2}^{-},...,{\hat{r}}_{6}^{-}\right)$ are determined as follows:

$$\begin{array}{l} A^{*}=\left(\Delta \left(0.0594\right),\;\Delta \left(0.0347\right),\;\Delta \left(0.0300\right),\;\Delta \left(0.1313\right),\;\Delta \left(0.1395\right),\;\Delta \left(0.0930\right)\right)\;,\;\\ A^{-}=\left(\Delta \left(0.1575\right),\;\Delta \left(0.0953\right),\;\Delta \left(0.1658\right),\;\Delta \left(0.0869\right),\;\;\Delta \left(0.0750\right),\;\Delta \left(0.0620\right)\right) \end{array}$$

It is now possible to develop different ITI-TOPSIS methods based on the ITIOWD operator in order to determine the most appropriate method to manage the HCW. 

In this example, we consider the maximum interval 2-tuple distance, the minimum interval 2-tuple distance, the ITNHD, the ITWHD, the ITOWD, the ITIOWD, the ITIOWED, the ITIOWGD, the ITIOWHD and the ITIOWCD operators. For convenience, we assume the following weighting vector $\omega ={\left(0.086,0.172,0.242,0.242,0.172,0.086\right)}^{T}$ in line with the normal distribution-based method [38]. The aggregated results are shown in Table 6 and Table 7.

It can be observed that, for most of the cases the best alternative is *A*_2_ because it seems to be the one with the highest closeness coefficient to the ideal alternative. However, for some particular cases, we may find another optimal choice. If we establish a ranking of the alternative HCW disposal technologies for each particular situation, we get the results as shown in Table 8. It may be mentioned here that the optimal choice would be the alternative with the highest value of $C_{i}^{*}$in each situation.

As can be seen, depending on the particular type of the interval 2-tuple induced distance operators used, the ranking of the HCW disposal alternatives may be different and thus the decision maker may make a different decision. The main advantages of using the ITI-TOPSIS method are that we can not only represent complex reordering processes in the decision making by using order inducing variables, but also consider different possible situations by using a wide range of particular distance operators. Therefore, the decision maker knows the results and optimal decisions that can be obtained with each particular case and select for his decision the one that is closest to his interest. In this example, it is clear that the best HCW treatment method is *A*_2_, although in some exceptional situations *A*_1_ or *A*_3_ could be optimal. 

To further validate the effectiveness of the proposed hybrid decision making approach based on interval 2-tuple induced distance operators and the modified TOPSIS method, the results of this study are compared with the ones obtained by the fuzzy VIKOR method [16] and the fuzzy MULTIMOORA method [18]. The ranking order of the four HCW disposal alternatives is $A_{2}≻A_{3}≻A_{1}≻A_{4}$ with the two listed methods, which is the same as the sequence of alternatives yielded via the ITI-TOPSIS method when six types of the interval 2-tuple induced distance operators are employed (*cf.* Table 8). Furthermore, the ranking results show that the first choice of HCW disposal technology remains the same, *i.e.*, A2, by the proposed approach and the methods of [16] and [18]. This demonstrates the validity of the presented ITI-TOPSIS method. But compared with the fuzzy logic-based methods, the new integrated MCDM framework using interval 2-tuple linguistic variables can deal with different types of assessment information provided by decision makers, and thus is more practical and more flexible for HCW treatment technology selection. Moreover, the proposed model has exact characteristic in linguistic information processing, which can effectively avoid the loss and distortion of information which occur formerly in the linguistic information processing.

## 5. Conclusions

The problem of medical waste requiring specialized treatment and management of its disposal is growing rapidly as a direct result of fast urbanization and population growth, especially in the developing world. In this paper, we proposed a hybrid decision making approach that uses interval 2-tuple induced distance operators and the TOPSIS method to solve the HCW treatment technology selection problem with linguistic information. Particularly, we introduced a new type of distance operator called the ITIOWD operator to calculate the separate measures of each alternative from the positive-ideal and the negative-ideal solutions. The ITI-TOPSIS method is able to model the uncertainty and diversity of assessment information offered by decision makers and can consider the complex attitudinal character of the decision maker in the decision process. Besides, it can provide much more complete information for decision making because it is able to consider a lot of different scenarios according to the interest of the decision maker. Finally, the feasibility and applicability of the proposed method have been illustrated with an empirical case study in Shanghai, China. The results showed that the proposed method for group decision making with interval 2-tuple linguistic information can effectively deal with the HCW disposal technology selection problem under uncertain and complex environments. 

This study has several implications for possible directions of further research. First, the weights of the selection criteria are given directly in this study. But, in many practical situations, it is normally difficult to determine the criteria weights subjectively. Therefore, in the future, it is suggested to develop an optimization model so as to derive the weighting vector of selection criteria objectively. Second, the criteria are assumed to be independent in the proposed method when modeling the HCW treatment technology selection problem. In actual cases, however, various types of relationships may exist among evaluation criteria. Accordingly, a modified model to deal with the interdependence among different criteria should be investigated in future research. In addition, we expect to consider the potential applications of the developed ITI-TOPSIS method to other decision making problems, such as material selection, factory location and personnel evaluation, to further validate its applicability and effectiveness. 

## Figures and Tables

**Table 1:** Linguistic assessments of the HCW treatment alternatives.

Decision Makers	Alternatives	Criteria					
		*C*_1_	*C*_2_	*C*_3_	*C*_4_	*C*_5_	*C*_6_
*DM*_1_	*A*_1_	M-H	M-H	H-VH	VH	H	M-H
	*A*_2_	H	L	VL-L	H	H	H
	*A*_3_	H	L	M	M-H	M-H	H
	*A*_4_	L-M	M-H	H	L-M	L	M-H
*DM*_2_	*A*_1_	M	M	MH-H	H	H	M-H
	*A*_2_	H	L-M	L	M-H	MH	H
	*A*_3_	MH	ML	ML	MH	VH	MH
	*A*_4_	L-M	M	M-H	M	M-MH	H
*DM*_3_	*A*_1_	L-M	L	H	MH-H	H	M-MH
	*A*_2_	H-VH	ML-M	VL	VH	MH	M
	*A*_3_	M	ML	L	H	H	M
	*A*_4_	ML	H	MH	MH	M-MH	H
*DM*_4_	*A*_1_	M	M-H	M-H	M-VH	M-H	M-H
	*A*_2_	H	VL	L	VH	M	M
	*A*_3_	M	L	L-M	M	M	M-H
	*A*_4_	L-M	M	M-H	H	L	H
*DM*_5_	*A*_1_	ML-M	ML-M	VH	H	H	MH-H
	*A*_2_	VH	ML	L-ML	H	M-H	M
	*A*_3_	M-H	L	ML-M	M-H	H	ML
	*A*_4_	MH	ML	VH	MH	MH	H

**Table 2:** Order inducing variables.

Alternatives	*C*_1_	*C*_2_	*C*_3_	*C*_4_	*C*_5_	*C*_6_
*A*_1_	22	13	14	27	18	16
*A*_2_	8	15	22	30	20	12
*A*_3_	12	6	19	14	8	10
*A*_4_	25	16	18	15	30	9

**Table 3:** Interval 2-tuple decision matrix.

Decision Makers	Alternatives	Criteria					
		*C*_1_	*C*_2_	*C*_3_	*C*_4_	*C*_5_	*C*_6_
*DM*_1_	*A*_1_	[(*a*_2_,0), (*a*_3_,0)]	[(*a*_2_,0), (*a*_3_,0)]	[(*a*_3_,0), (*a*_4_,0)]	[(*a*_4_,0), (*a*_4_,0)]	[(*a*_3_,0), (*a*_3_,0)]	[(*a*_2_,0), (*a*_3_,0)]
	*A*_2_	[(*a*_3_,0), (*a*_3_,0)]	[(*a*_1_,0), (*a*_1_,0)]	[(*a*_0_,0), (*a*_1_,0)]	[(*a*_3_,0), (*a*_3_,0)]	[(*a*_3_,0), (*a*_3_,0)]	[(*a*_3_,0), (*a*_3_,0)]
	*A*_3_	[(*a*_3_,0), (*a*_3_,0)]	[(*a*_1_,0), (*a*_1_,0)]	[(*a*_2_,0), (*a*_2_,0)]	[(*a*_2_,0), (*a*_3_,0)]	[(*a*_2_,0), (*a*_3_,0)]	[(*a*_3_,0), (*a*_3_,0)]
	*A*_4_	[(*a*_1_,0), (*a*_2_,0)]	[(*a*_2_,0), (*a*_3_,0)]	[(*a*_3_,0), (*a*_3_,0)]	[(*a*_1_,0), (*a*_2_,0)]	[(*a*_1_,0), (*a*_1_,0)]	[(*a*_2_,0), (*a*_3_,0)]
*DM*_2_	*A*_1_	[(*b*_3_,0), (*b*_3_,0)]	[(*b*_3_,0), (*b*_3_,0)]	[(*b*_4_,0), (*b*_5_,0)]	[(*b*_5_,0), (*b*_5_,0)]	[(*b*_5_,0), (*b*_5_,0)]	[(*b*_3_,0), (*b*_5_,0)]
	*A*_2_	[(*b*_5_,0), (*b*_5_,0)]	[(*b*_1_,0), (*b*_3_,0)]	[(*b*_1_,0), (*b*_1_,0)]	[(*b*_3_,0), (*b*_5_,0)]	[(*b*_4_,0), (*b*_4_,0)]	[(*b*_5_,0), (*b*_5_,0)]
	*A*_3_	[(*b*_4_,0), (*b*_4_,0)]	[(*b*_2_,0), (*b*_2_,0)]	[(*b*_2_,0), (*b*_2_,0)]	[(*b*_4_,0), (*b*_4_,0)]	[(*b*_6_,0), (*b*_6_,0)]	[(*b*_4_,0), (*b*_4_,0)]
	*A*_4_	[(*b*_1_,0), (*b*_3_,0)]	[(*b*_3_,0), (*b*_3_,0)]	[(*b*_3_,0), (*b*_5_,0)]	[(*b*_3_,0), (*b*_3_,0)]	[(*b*_3_,0), (*b*_4_,0)]	[(*b*_5_,0), (*b*_5_,0)]
*DM*_3_	*A*_1_	[(*c*_2_,0), (*c*_4_,0)]	[(*c*_2_,0), (*c*_2_,0)]	[(*c*_6_,0), (*c*_6_,0)]	[(*c*_5_,0), (*c*_6_,0)]	[(*c*_6_,0), (*c*_6_,0)]	[(*c*_4_,0), (*c*_5_,0)]
	*A*_2_	[(*c*_6_,0), (*c*_7_,0)]	[(*c*_3_,0), (*c*_4_,0)]	[(*c*_1_,0), (*c*_1_,0)]	[(*c*_7_,0), (*c*_7_,0)]	[(*c*_5_,0), (*c*_5_,0)]	[(*c*_4_,0), (*c*_4_,0)]
	*A*_3_	[(*c*_4_,0), (*c*_4_,0)]	[(*c*_3_,0), (*c*_3_,0)]	[(*c*_2_,0), (*c*_2_,0)]	[(*c*_6_,0), (*c*_6_,0)]	[(*c*_6_,0), (*c*_6_,0)]	[(*c*_4_,0), (*c*_4_,0)]
	*A*_4_	[(*c*_3_,0), (*c*_3_,0)]	[(*c*_6_,0), (*c*_6_,0)]	[(*c*_5_,0), (*c*_5_,0)]	[(*c*_5_,0), (*c*_5_,0)]	[(*c*_4_,0), (*c*_5_,0)]	[(*c*_6_,0), (*c*_6_,0)]
*DM*_4_	*A*_1_	[(*a*_2_,0), (*a*_2_,0)]	[(*a*_2_,0), (*a*_3_,0)]	[(*a*_2_,0), (*a*_3_,0)]	[(*a*_2_,0), (*a*_4_,0)]	[(*a*_2_,0), (*a*_3_,0)]	[(*a*_2_,0), (*a*_3_,0)]
	*A*_2_	[(*a*_3_,0), (*a*_3_,0)]	[(*a*_0_,0), (*a*_0_,0)]	[(*a*_1_,0), (*a*_1_,0)]	[(*a*_4_,0), (*a*_4_,0)]	[(*a*_2_,0), (*a*_2_,0)]	[(*a*_2_,0), (*a*_2_,0)]
	*A*_3_	[(*a*_2_,0), (*a*_2_,0)]	[(*a*_1_,0), (*a*_1_,0)]	[(*a*_1_,0), (*a*_2_,0)]	[(*a*_2_,0), (*a*_2_,0)]	[(*a*_2_,0), (*a*_2_,0)]	[(*a*_2_,0), (*a*_3_,0)]
	*A*_4_	[(*a*_1_,0), (*a*_2_,0)]	[(*a*_2_,0), (*a*_2_,0)]	[(*a*_2_,0), (*a*_3_,0)]	[(*a*_3_,0), (*a*_3_,0)]	[(*a*_1_,0), (*a*_1_,0)]	[(*a*_3_,0), (*a*_3_,0)]
*DM*_5_	*A*_1_	[(*b*_2_,0), (*b*_3_,0)]	[(*b*_2_,0), (*b*_3_,0)]	[(*b*_6_,0), (*b*_6_,0)]	[(*b*_5_,0), (*b*_5_,0)]	[(*b*_5_,0), (*b*_5_,0)]	[(*b*_4_,0), (*b*_5_,0)]
	*A*_2_	[(*b*_6_,0), (*b*_6_,0)]	[(*b*_2_,0), (*b*_2_,0)]	[(*b*_1_,0), (*b*_2_,0)]	[(*b*_5_,0), (*b*_5_,0)]	[(*b*_3_,0), (*b*_5_,0)]	[(*b*_3_,0), (*b*_3_,0)]
	*A*_3_	[(*b*_3_,0), (*b*_5_,0)]	[(*b*_1_,0), (*b*_1_,0)]	[(*b*_2_,0), (*b*_3_,0)]	[(*b*_3_,0), (*b*_5_,0)]	[(*b*_5_,0), (*b*_5_,0)]	[(*b*_2_,0), (*b*_2_,0)]
	*A*_4_	[(*b*_4_,0), (*b*_4_,0)]	[(*b*_2_,0), (*b*_2_,0)]	[(*b*_6_,0), (*b*_6_,0)]	[(*b*_4_,0), (*b*_4_,0)]	[(*b*_4_,0), (*b*_4_,0)]	[(*b*_5_,0), (*b*_5_,0)]

**Table 4:** Collective interval 2-tuple decision matrix.

Alternatives	*C*_1_	*C*_2_	*C*_3_	*C*_4_	*C*_5_	*C*_6_
*A*_1_	∆[0.408, 0.538]	∆[0.408, 0.525]	∆[0.696, 0.829]	∆[0.713, 0.875]	∆[0.713, 0.775]	∆[0.517, 0.738]
*A*_2_	∆[0.792, 0.829]	∆[0.217, 0.321]	∆[0.150, 0.204]	∆[0.808, 0.875]	∆[0.608, 0.642]	∆[0.604, 0.604]
*A*_3_	∆[0.571, 0.604]	∆[0.296, 0.296]	∆[0.313, 0.392]	∆[0.608, 0.679]	∆[0.708, 0.746]	∆[0.554, 0.617]
*A*_4_	∆[0.313, 0.479]	∆[0.558, 0.596]	∆[0.625, 0.754]	∆[0.579, 0.617]	∆[0.417, 0.488]	∆[0.738, 0.775]

**Table 5:** Weighted collective interval 2-tuple decision matrix.

Alternatives	*C*_1_	*C*_2_	*C*_3_	*C*_4_	*C*_5_	*C*_6_
*A*_1_	∆[0.0776, 0.1021]	∆[0.0653, 0.0840]	∆[0.1392, 0.1658]	∆[0.1069, 0.1313]	∆[0.1283, 0.1395]	∆[0.0620, 0.0885]
*A*_2_	∆[0.1504, 0.1575]	∆[0.0347, 0.0513]	∆[0.0300, 0.0408]	∆[0.1213, 0.1313]	∆[0.1095, 0.1155]	∆[0.0725, 0.0725]
*A*_3_	∆[0.1085, 0.1148]	∆[0.0473, 0.0473]	∆[0.0625, 0.0783]	∆[0.0913, 0.1019]	∆[0.1275, 0.1343]	∆[0.0665, 0.0740]
*A*_4_	∆[0.0594, 0.0910]	∆[0.0893, 0.0953]	∆[0.1250, 0.1508]	∆[0.0869, 0.0925]	∆[0.0750, 0.0878]	∆[0.0885, 0.0930]

**Table 6:** Aggregated results 1.

Distance Operators		*A*_1_	*A*_2_	*A*_3_	*A*_4_
Max	$S_{i}^{+}$	∆[0.1225]	∆[0.0946]	∆[0.0523]	∆[0.1079]
	$S_{i}^{-}$	∆[0.0677]	∆[0.1304]	∆[0.0954]	∆[0.0823]
	$C_{i}^{*}$	∆[0.3559]	∆[0.5796]	∆[0.6462]	∆[0.4328]
Min	$S_{i}^{+}$	∆[0.0056]	∆[0.0050]	∆[0.0086]	∆[0.0023]
	$S_{i}^{-}$	∆[0.0133]	∆[0.0036]	∆[0.0082]	∆[0.0028]
	$C_{i}^{*}$	∆[0.7020]	∆[0.4161]	∆[0.4889]	∆[0.5556]
ITNHD	$S_{i}^{+}$	∆[0.0381]	∆[0.0268]	∆[0.0286]	∆[0.0472]
	$S_{i}^{-}$	∆[0.0343]	∆[0.0456]	∆[0.0439]	∆[0.0252]
	$C_{i}^{*}$	∆[0.4741]	∆[0.6298]	∆[0.6056]	∆[0.3479]
ITWHD	$S_{i}^{+}$	∆[0.0446]	∆[0.0185]	∆[0.0283]	∆[0.0576]
	$S_{i}^{-}$	∆[0.0317]	∆[0.0578]	∆[0.0480]	∆[0.0186]
	$C_{i}^{*}$	∆[0.4152]	∆[0.7574]	∆[0.6290]	∆[0.2440]
ITOWD	$S_{i}^{+}$	∆[0.0317]	∆[0.0211]	∆[0.0283]	∆[0.0462]
	$S_{i}^{-}$	∆[0.0322]	∆[0.0409]	∆[0.0429]	∆[0.0211]
	$C_{i}^{*}$	∆[0.5040]	∆[0.6597]	∆[0.6029]	∆[0.3133]

**Table 7:** Aggregated results 2.

Distance Operators		*A*_1_	*A*_2_	*A*_3_	*A*_4_
ITIOWD	$S_{i}^{+}$	∆[0.0365]	∆[0.0216]	∆[0.0302]	∆[0.0551]
	$S_{i}^{-}$	∆[0.0359]	∆[0.0497]	∆[0.0367]	∆[0.0251]
	$C_{i}^{*}$	∆[0.4964]	∆[0.6972]	∆[0.5490]	∆[0.3132]
ITIOWED	$S_{i}^{+}$	∆[0.0545]	∆[0.0323]	∆[0.0341]	∆[0.0652]
	$S_{i}^{-}$	∆[0.0427]	∆[0.0639]	∆[0.0454]	∆[0.0379]
	$C_{i}^{*}$	∆[0.4393]	∆[0.6643]	∆[0.5714]	∆[0.3672]
ITIOWGD	$S_{i}^{+}$	∆[0.0214]	∆[0.0142]	∆[0.0253]	∆[0.0385]
	$S_{i}^{-}$	∆[0.0282]	∆[0.0332]	∆[0.0256]	∆[0.0117]
	$C_{i}^{*}$	∆[0.5695]	∆[0.7009]	∆[0.5034]	∆[0.2327]
ITIOWHD	$S_{i}^{+}$	∆[0.0137]	∆[0.0104]	∆[0.0204]	∆[0.0164]
	$S_{i}^{-}$	∆[0.0224]	∆[0.0181]	∆[0.0172]	∆[0.0059]
	$C_{i}^{*}$	∆[0.6202]	∆[0.6360]	∆[0.4578]	∆[0.2656]
ITIOWCD	$S_{i}^{+}$	∆[0.0690]	∆[0.0430]	∆[0.0370]	∆[0.0725]
	$S_{i}^{-}$	∆[0.0475]	∆[0.0757]	∆[0.0517]	∆[0.0469]
	$C_{i}^{*}$	∆[0.4079]	∆[0.6381]	∆[0.5829]	∆[0.3931]

**Table 8:** Ranking of the alternatives.

Distance Operators	Ranking	Distance Operators	Ranking
Max	$A_{3}≻A_{2}≻A_{4}≻A_{1}$	ITIOWD	$A_{2}≻A_{3}≻A_{1}≻A_{4}$
Min	$A_{1}≻A_{4}≻A_{3}≻A_{2}$	ITIOWED	$A_{2}≻A_{3}≻A_{1}≻A_{4}$
ITNHD	$A_{2}≻A_{3}≻A_{1}≻A_{4}$	ITIOWGD	$A_{2}≻A_{1}≻A_{3}≻A_{4}$
ITWHD	$A_{2}≻A_{3}≻A_{1}≻A_{4}$	ITIOWHD	$A_{2}≻A_{1}≻A_{3}≻A_{4}$
ITOWD	$A_{2}≻A_{3}≻A_{1}≻A_{4}$	ITIOWCD	$A_{2}≻A_{3}≻A_{1}≻A_{4}$
